# Cornea opacity, uveitis with iris atrophy and lens damage following cosmetic high-intensity ultrasound of the eyelid: a case report

**DOI:** 10.1186/s12886-023-02947-9

**Published:** 2023-05-22

**Authors:** Samara Barbara Marafon, Diane Ruschel Marinho, Sérgio Kwitko

**Affiliations:** 1 Hospital de Clinicas de Porto Alegre, 2350 Ramiro Barcelos Porto Alegre, Porto Alegre, RS 90035-903 Brazil; 2 Oftalmocentro, Porto Alegre, RS Brazil

**Keywords:** Cornea opacity, Lens opacity, Microfocused ultrasound, Facial rejuvenation

## Abstract

**Background:**

High-intensity focused ultrasound (HIFU) is a cosmetic procedure that aims to tone the skin through thermal collagen coagulation. The energy is delivered in the deep layers of the skin, and because of these characteristics, the risks of severe damage to adjacent tissue and the ocular surface may be underestimated. Previous reports have demonstrated superficial corneal opacities, cataracts, increased intraocular pressure, or ocular refractive changes in different patients following HIFU. In this case, we report deep stromal opacities associated with anterior uveitis, iris atrophy and lens opacity formation following a single HIFU superior eyelid application.

**Case presentation:**

A 47-year-old female presented to the ophthalmic emergency department complaining of pain, hyperemia and photophobia in the right eye following a HIFU application to the superior right eyelid. A slit lamp examination showed three temporal-inferior corneal infiltrates with edema and severe anterior uveitis. The patient was treated with topical corticosteroids, and six months later, there was residual corneal opacity, iris atrophy and peripherical cataract formation. No surgical procedure was needed, and the final vision was Snellen 20/20 (1.0).

**Conclusion:**

The risk of severe impairment to the ocular surface and ocular tissues may be underestimated. Cosmetic surgeons and ophthalmologists must be aware of the complications, and the long-term follow-up of these changes needs further investigation and discussion. Safety protocols of the HIFU intensity threshold for thermal lesions in the eye and the use of protective eye devices should be better evaluated.

## Background

High-intensity focused ultrasound (HIFU) is a relatively new cosmetic procedure that uses non-invasive ultrasound technology to induce thermal collagen coagulation to tone the skin. This technique works differently from conventional cosmetic lasers, which act on the outer layers of the skin. HIFU energy creates thermal injury in the deep layers of the skin in musculoaponeurotic systems, destroys the intermolecular alpha crosslinks of collagen and induces collagen shrinkage [1]. Because of these characteristics, the risk of severe damage to other tissues or the ocular surface may be underestimated. Nonetheless, previous reports demonstrating superficial corneal opacities, cataracts, increased intraocular pressure, or ocular refractive changes involving different patients have been published [2–5]. In this case, we report deep stromal opacities associated with anterior uveitis, iris atrophy and lens opacity formation following a single HIFU superior eyelid application. The long-term follow-up of these changes is not yet known and needs further investigation and discussion.

## Case presentation

A 47-year-old female without any ophthalmic history presented to the ophthalmic emergency department complaining of pain, hyperemia and photophobia in the right eye (OD). The symptoms started during a cosmetic procedure of HIFU (Ultraformer, Medsystems, Brazil) around the right upper eyelid area. The treatment was put on hold as the symptoms started, and the patient did not receive the application to the left eyelid. The procedure was performed by a trained dermatologist without any eye protection device. More detailed information about the probe and the power was not available.

**Fig. 1:**
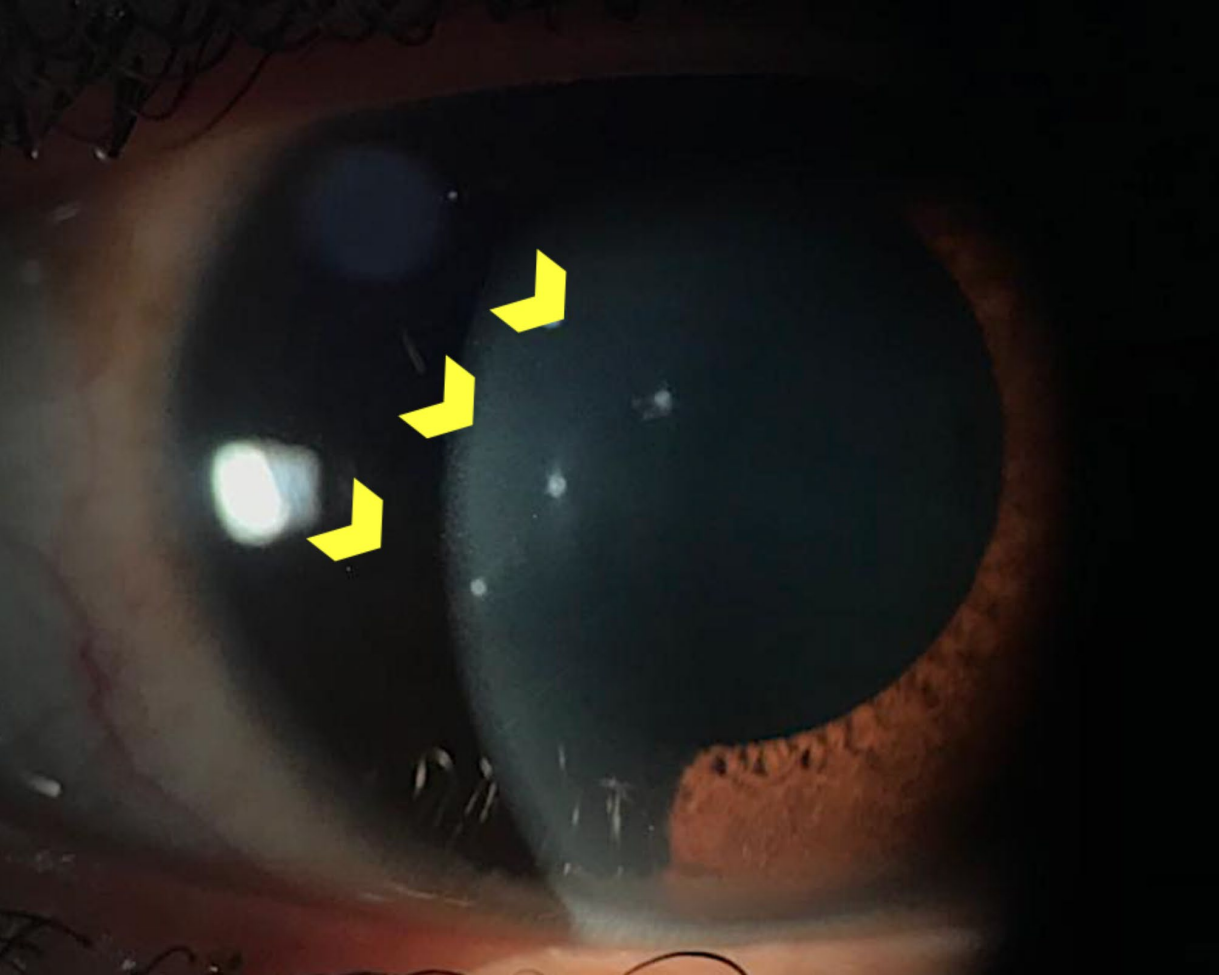
Initial presentation with three stromal corneal infiltrates and edema

The initial uncorrected visual acuity was 20/30 OD. Biomicroscopy showed mild ocular hyperemia and a cornea with three punctiform epithelium defects with stromal infiltrate and local stromal edema, with an adjacent clear cornea (Fig. 1). The intraocular pressure (IOP) was 16 mmHg in the OD. The anterior chamber showed anterior chamber cels 4/4+, an iris without lesions, and a transparent lens. Fundoscopy showed no abnormalities. The patient was treated with prednisolone 10 mg/ml (Predfort, Allergan, Brazil) eye drops QID in the first week and then tapered off until the completion of 30 days of treatment. The patient was referred to a cornea specialist and, after 5 days of treatment, the symptoms smoothly regressed, and the IOP remained at 16 mmHg.

**Fig. 2:**
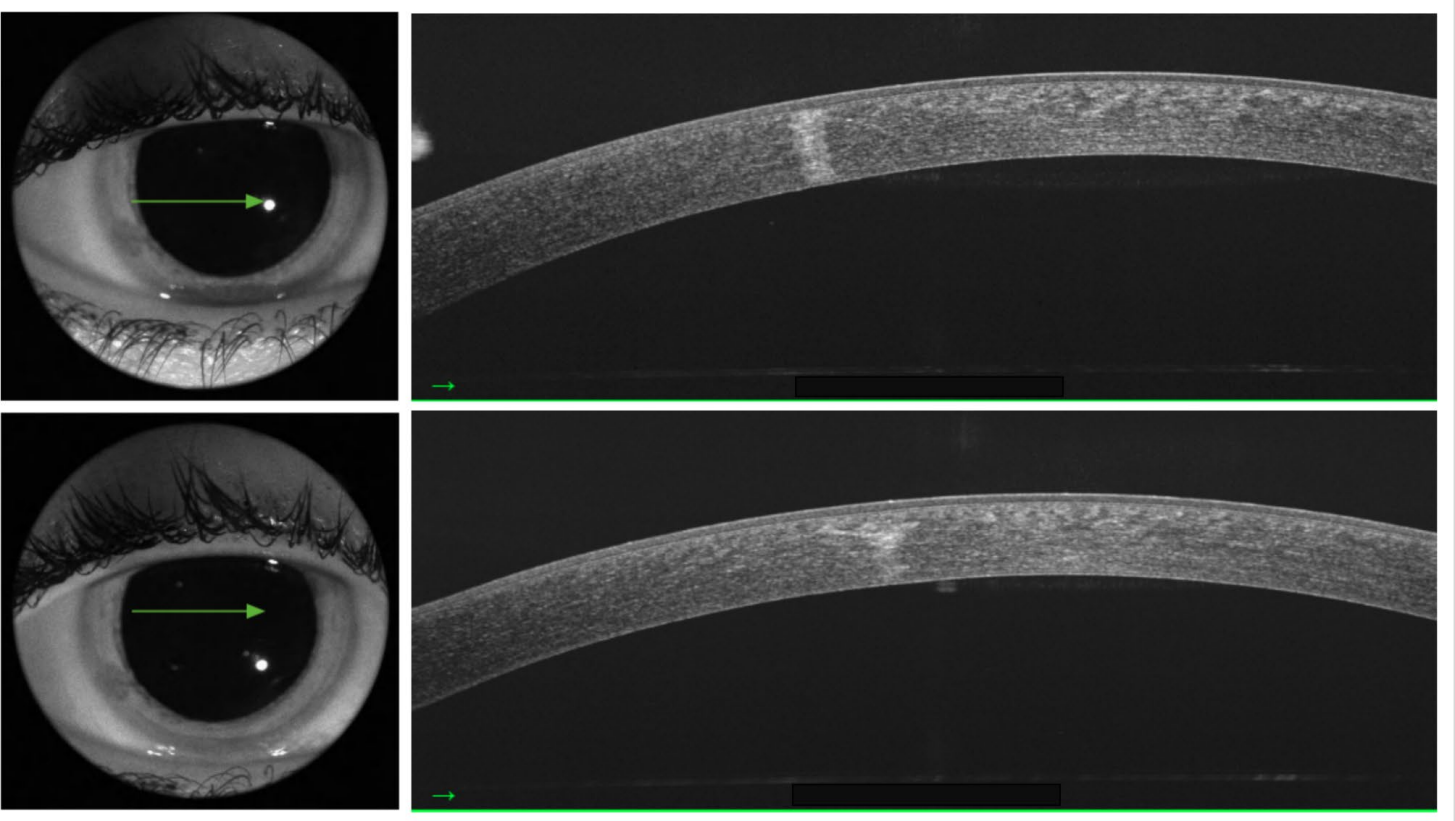
Corneal optical coherence tomography (OCT) showing corneal stromal opacity on day 21. The epithelium was healed, and the endothelium was not affected

**Fig. 3:**
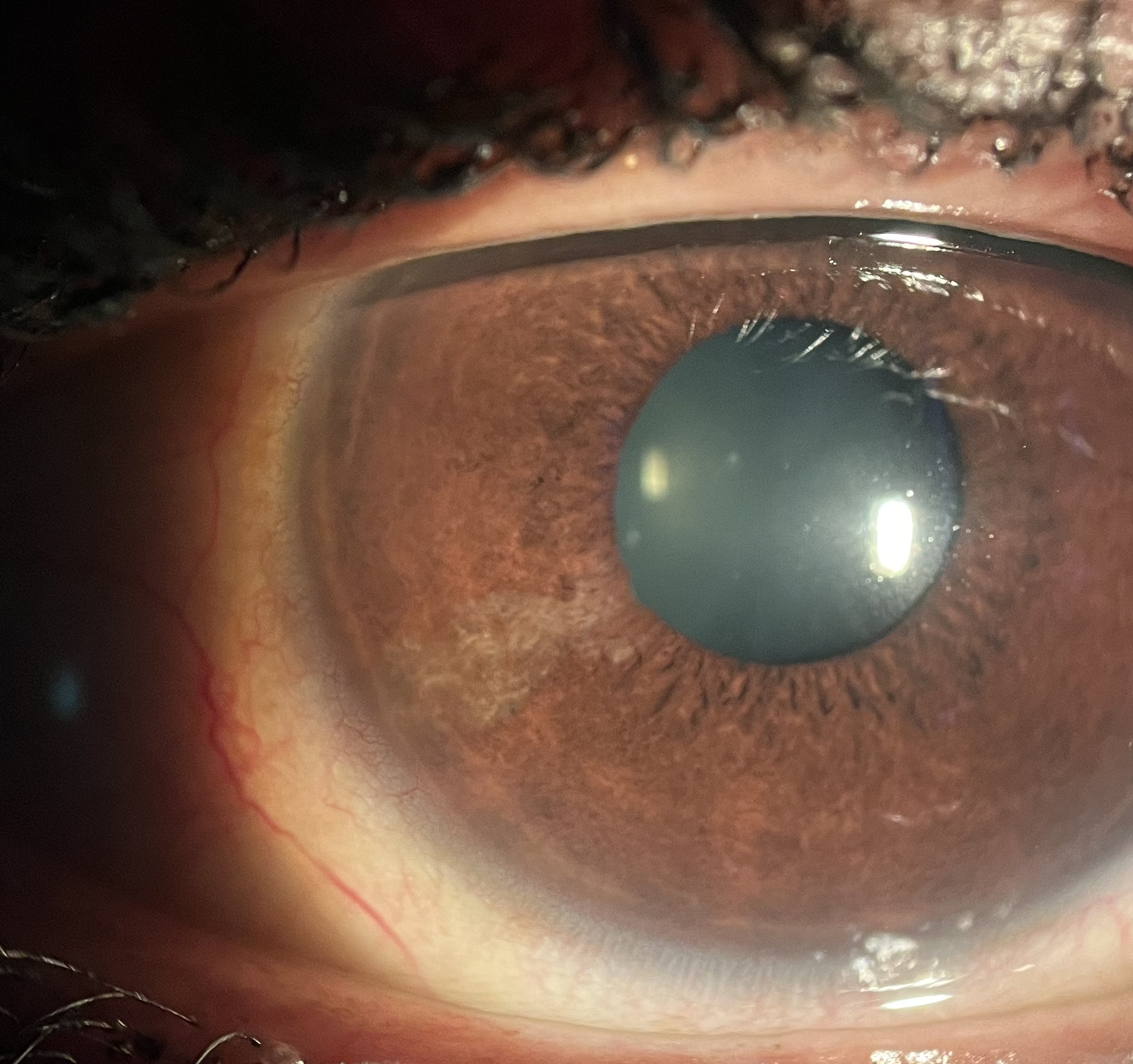
Slip lamp biomicroscopy showing the remaining corneal opacity and iris atrophy at 6 months after the HIFU application

**Fig. 4:**
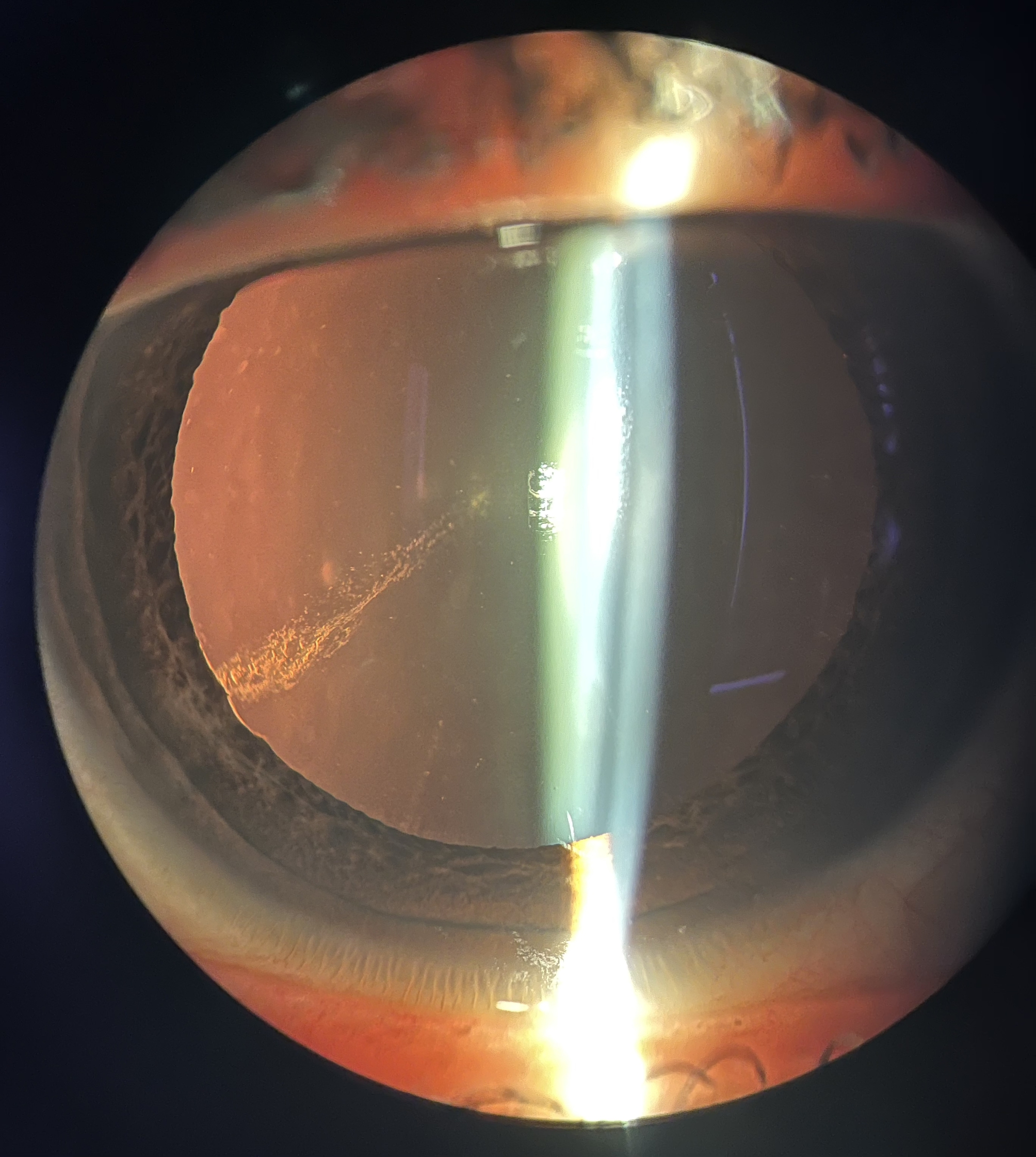
Slip lamp biomicroscopy showing lens opacity (anterior cortical cataract) in the inferior temporal quadrant at 6 months after the HIFU application

After 21 days, the patient presented a full regression of symptoms, with uncorrected visual acuity of 20/20 (1.0) in the OD, slip-lamp biomicroscopy with a nonhyperemic eye, and an uninflamed anterior chamber. In the cornea, a healed epithelium with three stromal opacities remained, extending through the entire thickness of the stroma without edema. The opacity extension was registered on corneal Optical coherence tomography (OCT) (Fig. 2). Corneal topography was slightly irregular but symmetrical, and no flattening or steepening was seen in area of the opacity. At the six-month appointment, the patient had no complaints; however, biomicroscopy showed iris atrophy (Fig. 3) and lens opacity (Fig. 4) in the inferior temporal quadrant, coincident with the area of initial and residual corneal opacity. The cataract was anterior cortical and did not reach the visual axis. No treatment was required so far, and her vision remained at 20/20 (1.0) OD. The unharmed left eye remained at 20/20 at all time points.

## Discussion and conclusion

Ultrasound has diagnostic and therapeutic applications in medicine and ophthalmology. At high intensity, ultrasound can produce significant biological effects through cavitation and thermal mechanisms. Ultrasound can be delivered through specially designed transducers, and in ophthalmology, it can be used to cause ciliodestruction, treat tumors, and treat other retinal and lens pathologies, although it requires significant improvements [6]. Conversely, the use of HIFU in cosmetic and dermatological procedures has been widely established, and coagulation of the deep layers of the skin is used to create facial contour and a rejuvenescent effect.

The HIFU transducers generate ultrasound energy at a frequency of 4 to 7 MHz, which is focused on yielding discrete zones of thermal microcoagulation spaced 1.0 to 1.5 mm apart along defined tissue planes within the dermis and hypodermis [7]. According to the energy settlement, the device can deliver energy to a tissue depth of 3 to 4.5 mm. The eyelid is the thinnest skin on the body, and the exact HIFU targeting for safe energy intervals and distance from the orbital margins are complex. For lifting of the eyebrows, the device should be applied to the lateral part of the forehead, including the region just above the lateral two-thirds of the eyebrows, with energy set to a depth of 3 mm [7]. Furthermore, its direct application on the regular position of the superior eyelid is not recommended. The technique requires an upward dislocation of the superior eyelid skin in the direction of the orbital rim. We did not witness the application, but the very specific characteristic of the findings – e.g. the regular and aligned corneal opacity that is very similar to the HIFU probe – suggest that unintentional energy must have been delivered directly to the superior eyelid without any skin dislocation.

In this case, we described a patient with multiple anterior segment abnormalities due to a single application of HIFU in the superior eyelid. The corneal infiltrates might result from two concomitant processes: collagen shrinkage due to its direct coagulation and the consequence of the inflammatory reaction with persistent myofibroblast transformation. These myofibroblast transformations occur when defects in the epithelial basement membrane allow penetration of proinflammatory factors such as transforming growth factor-beta (TGFβ) and platelet-derived growth factor (PDGF) derived from the epithelium to penetrate the stroma [8]. They produce a disorganized and excessive extracellular matrix that leads to corneal opacity. The direct collagen shrinkage can be explained by the ultrasound mechanism: its waves induce vibration in the composite molecules of the tissue, and the friction between the molecules generates heat. Heat acts in Type I collagen, which is composed of a triple-helical conformation with alpha and beta cross-links and shrinks the intermolecular alpha cross-link. Collagen shrinkage in the cornea results in a density change and corneal opacity. It has previously been shown that after HIFU application, corneal lesions reveal more extensive disruption, including the formation of a superficial stromal depression and a larger zone of edema and disorganization surrounding each lesion [9].

Iris defects and ciliary spasms have been previously reported, and might seem definitive due to direct thermal coagulation of the tissue [5]. Cataract formation in the same topography of corneal and iris defects suggests that the energy must have reached as deep as the lens. As shown in an experimental model with HIFU by Lizzi et al. [10], the heat created by the focused ultrasound denatured proteins inside the lens by thermocoagulation, forming dense opacities. They studied the correlation between intensity and exposure and reported that the formation of cataracts was possible during exposure times as short as 100 msec. Cataracts have also been described following a single application of ultrasound cycloplasty for glaucoma [11]. To date, the cataract opacity in our patient has not reached the visual axis and has not required complementary treatment.

Ultimately, the remaining opacities did not negatively impact visual acuity, and the patient is in regular follow-up. Early topical steroid eye drops – same day, few hours after the application – might have relieved excessive wound contracture due to collagen shrinkage. Steroids are anti-inflammatory agents that inhibit the immune response and collagen synthesis and might have played a role in halting the process.

Although our patient has remained stable without vision loss during the six-month follow-up, the risks of HIFU damage in the eye can be very tricky and sight threatening. The exact HIFU intensity for thermal lesions in the eye and their irreversibility should be better evaluated, and the procedure should be put on hold until further safety studies are performed. Ocular protection gear should be considered for all patients.

## Data Availability

All data generated or analysed during this study are included in this published article.

## Abbreviations

HIFU: high-intensity focused ultrasound
IOP: intraocular pressure
OD: right eye
QID: four times a day
TGFβ: transforming growth factor-beta
PDGF: platelet-derived growth factor
